# Experiences of Nursing Students during the Abrupt Change from Face-to-Face to e-Learning Education during the First Month of Confinement Due to COVID-19 in Spain

**DOI:** 10.3390/ijerph17155519

**Published:** 2020-07-30

**Authors:** Antonio Jesús Ramos-Morcillo, César Leal-Costa, José Enrique Moral-García, María Ruzafa-Martínez

**Affiliations:** 1 Faculty of Nursing, University of Murcia, 30100 Murcia, Spain; ajramos@um.es (A.J.R.-M.); maruzafa@um.es (M.R.-M.); 2 Physical Activity and Sports Sciences, Faculty of Education, Pontifical University of Salamanca, Street Henry Collet, 52-70, 37007 Salamanca, Spain

**Keywords:** COVID-19, pandemics, students, nursing, teaching, education, distance, schools, Life Changing Events, qualitative research

## Abstract

The current state of alarm due to the COVID-19 pandemic has led to the urgent change in the education of nursing students from traditional to distance learning. The objective of this study was to discover the learning experiences and the expectations about the changes in education, in light of the abrupt change from face-to-face to e-learning education, of nursing students enrolled in the Bachelor’s and Master’s degree of two public Spanish universities during the first month of confinement due to the COVID-19 pandemic. Qualitative study was conducted during the first month of the state of alarm in Spain (from 25 March–20 April 2020). Semi-structured interviews were given to students enrolled in every academic year of the Nursing Degree, and nurses who were enrolled in the Master’s programs at two public universities. A maximum variation sampling was performed, and an inductive thematic analysis was conducted. The study was reported according with COREQ checklist. Thirty-two students aged from 18 to 50 years old participated in the study. The interviews lasted from 17 to 51 min. Six major themes were defined: (1) practicing care; (2) uncertainty; (3) time; (4) teaching methodologies; (5) context of confinement and added difficulties; (6) face-to-face win. The imposition of e-learning sets limitations for older students, those who live in rural areas, with work and family responsibilities and with limited electronic resources. Online education goes beyond a continuation of the face-to-face classes. Work should be done about this for the next academic year as we face an uncertain future in the short-term control of COVID-19.

## 1. Introduction

The fast propagation of the severe acute respiratory syndrome coronavirus 2 (SARS-CoV-2) led to its definition as a pandemic on 13 March 2020 by the WHO [1], as it met the epidemiological criteria and had infected more than 100,000 people in 100 countries [2,3,4]. The main public health recommendation was to remain at home and stay safe within it [5]. The world, in a globalized manner, is facing an extraordinary public health emergency in which the nurses are, as always, on the front line. Challenges are even greater in this period of pandemic [6,7], and nurses have the knowledge and aptitudes for providing the care necessary in the different clinical scenarios [5] that are emerging.

However, this pandemic is not only affecting the area of health. A great part of the nursing activity is affected as well. In Spain, as well as in other countries, the presence of nursing students in health care centers has been suspended [5]. It has been observed how, at great speeds, schools and universities have closed in the world, affecting more than 1.570 million students in 191 countries [8]. It has been necessary to decide how to continue the education of future nurses, and multiple education solutions have been deployed, all of which are based on distance learning. The professors, experts in the subjects and knowledgeable about the didactics of traditional classes, have found themselves compelled to deal with e-learning overnight, although not all of them were prepared. The same has occurred with the students, who have had to change from a model based on obligations and face-to-face learning, to a model in which the students will have to freely and voluntarily become involved in their learning [9]. All of this aside from finding themselves in a context of expectation and uncertainty.

Nursing educators (teachers, managers) must guarantee that the students meet the academic requirements, and at the same time, recognize the current conditions faced by the health services and the needs of simultaneously satisfying the demands from students, parents, brothers, partners, and the multiple roles every individual play in their day-to-day lives. Internationally and locally, a great variety of criteria for learning and evaluation, etc., have appeared, which are adapted to their national, work and social contexts. For example, and with respect to evaluation, in Berkeley (California, USA), a pass/fail grading has been proposed [10], and in Spain, the Association of Spanish Universities (CRUE) has recommended adapting the evaluation tests utilizing distance learning evaluation procedures [11].

Understanding the experiences and expectations of the students when faced with this important change, is necessary for helping the education and teaching authorities to assign sufficient resources and re-orient university education for nursing students. To be able to manage this situation in an imminent future, it is necessary to learn from these experiences and to define the strong and weak points. The objective of this study was to discover the learning experiences and the expectations about the changes in education of nursing students enrolled in the Bachelor’s and Master’s nursing degrees in two Spanish public universities, when faced with the abrupt change from face-to-face to e-learning education during the first month of confinement due to the COVID-19 pandemic.

## 2. Materials and Methods

### 2.1. Study Design

A qualitative approach was utilized, and an inductive thematic analysis [12] was conducted to understand the experiences and expectations of the participants.

### 2.2. Settings and Participants

This research study was conducted in its entirety during the first month of the state of alarm in Spain (which began on 14 March 2020). The state of alarm implied the confinement of the entire population, the closing of all the schools and universities, closing of non-essential businesses, closing of borders and ceasing all non-essential activities. The people were only allowed to go out to the street for essential matters: shopping of food, going to pharmacies, banks, and to care for older people who were dependent, etc.

In Spain, Bachelor’s degree in Nursing has a duration of four years (240 European Credit Transfer System, ECTS) and it is common for a Master’s degree to have a duration of one year (60 ECTS). The reference population in this study was students from every academic year in the Bachelor´s degree in Nursing, or nurses who were conducting their Master’s studies, enrolled in universities in Murcia and Granada (Spain). The participants were selected through the use of a maximum variation sampling strategy [13] to obtain heterogeneous and rich information that represented the main sociodemographic variables: gender, age, academic year, rural/urban, children, Bachelor’s/Master’s, university of Murcia and Granada. The maximum variation strategy is utilized to find the greatest diversity of discourses possible to identify and analyze the largest volume possible of expressions/presentation of the phenomenon studied to explain conditions/contexts where each one of them takes place.

If one did not answer the request, the students themselves proposed a replacement with another participant with similar characteristics. None of the students contacted disagreed to participate.

The students were invited to participate through the Student Delegation at the university, utilizing snowball sampling. This technique allowed us to build the sample by asking each interviewee for suggestions of people who had a similar or different perspective. This is an approach for locating information-rich key informants [13]. The saturation criterion was applied to establish the number of informants needed, an accepted method to estimate the sample size [14].

### 2.3. Data Collection

Semi-structured interviews were conducted to obtain the information. The semi-structured interview is normally based on a script, where the subject matter and part of the questions have been planned before starting, but it also offers the possibility of changing or adding new questions as the interview and/or the research study moves forward, with new interviews conducted. It is the most common type of interview utilized in qualitative research on health. Data were collected from 25 March to 20 April 2020. This was done in the first month as it the period of time with the greatest cognitive and social impact on learning and to obtain results that could be used to support, or not, the education measures that were utilized. All the interviews were individual and were performed online through electronic resources after agreeing on a day and time. The interviews were recorded and notes were made after each interview. All the interviews were conducted by researchers who had sufficient training and experience in semi-structured interviews (A.J.R.-M., M.R.-M.). The interviewers did not have an academic relationship with the informants. The interview followed a script which shifted from general to specific matters, and dealt with general aspects of the confinement, teaching methodologies utilized, learning and expectations (Table A1). A prior pilot study of the script was conducted [15].

### 2.4. Data Analysis

The 6 phases proposed for the thematic analysis were followed [16]: (1) Familiarizing yourself with your data; (2) Generating initial codes; (3) Searching for themes; (4) Reviewing themes; (5) Defining and naming themes; (6) Producing the report. The recorded interviews were transcribed verbatim. Once transcribed, the interviews were imported to the MAXQDA 12 program for its posterior analysis. A.J.R.-M., M.R.-M., C.L.-C. and J.E.M.-G. coded the data. The transcriptions, coding and themes-subthemes were discussed by the research team for their verification. Finally, participants provided feedback on the findings. The study was reported according to the Consolidated Criteria for Reporting Qualitative Research (COREQ) [15].

### 2.5. Ethical Considerations

This research study was approved by the Research Ethics Commission from the University of Murcia (ID: 2800/2020). All the participants received an informational electronic document about the purpose and research process, which they later kept. They were advised that their participation was voluntary. They could ask and reflect prior to the interview. Each participant was given a code to maintain anonymity. 

## 3. Results

A total of 32 interviews were conducted, and they lasted between 17 and 51 min. The shortest interviews corresponded to the more advanced academic years (3rd and 4th year students). Of these participants, 75% were women and 25% men. The age of the participants oscillated between 18–50 years old, with an average age of 25.3, and with a participation rate of 69% for the students from the University of Murcia, and 21.8% for the University of Granada students. The sample was composed by 18.75% of the students enrolled in their 1st or 2nd academic year or in the Master’s program, which accounted for about 57% of the sample, and 21.8% from the 3rd and 4th academic years, for a total of about 43%. Of those interviewed, 21.8% had children and 21.8% lived in a rural setting. Some of the characteristics of the participants are found in Table 1. 

Six major themes were defined: (1) practicing the nursing care; (2) uncertainty; (3) time; (4) teaching methodologies; (5) the context of confinement and the added difficulties; (6) face-to-face education win. A detailed description of the themes and sub-themes can be found in Table 2.

### 3.1. Practicing Nursing Care

The outstandingly practical component of care in nursing education was the most emotional aspect for the students. The experiences found were differentiated according to the group of students, depending if they had or not practice-based subjects during the education period affected by the state of alarm, the proximity to ending their training as nurses, or if they were health professionals who were conducting post-graduate studies.

#### 3.1.1. The Value of Clinical Training

For 1st and 2nd year students, the learning is normally done with courses that are eminently theoretical or theory/practical. The informants indicated that this transitory e-learning will not have a special influence on their training, as long as all the clinical training on health care institutions is present:

“In think that it’s not something that will affect us excessively for good or bad. In my year [1st]. In other years it will, because they have clinical training” P5

By contrast, 3rd and 4th year students whose coursework is mainly based on clinical training in health care institutions placed value on clinical training. They linked it with the acquisition of competences and referred to it as being an essential part of health sciences degrees:

“My education would not be good if clinical training was missing” P15; “without the clinical training, we can’t acquire competences” P19; “Especially in our degree, the clinical training …” P21

Clinical training provides them with security in the learning of nursing care in health care services. Part of the students in their last year (4th year) indicated that they would rather not graduate in July to do all the clinical training, therefore graduating later:

“I don’t feel prepared. My Erasmus in Italy was really bad because I was a nursing student and a foreigner. At the hospital, I don’t feel confident” P10; “Some of us prefer not to graduate in June and to do the clinical training” P14

The Master’s students indicated that not being able to do the clinical training implied the loss of job opportunities:

“If you cannot do the clinical training, you will lose job opportunities” P22

#### 3.1.2. To Help

All the participants expressed their wish to help during the pandemic. They expressed their desire to be nurses to help. At the University of Granada, a list of volunteers in their 4th year was even created. The expectation was present that the government could mobilize them in case of need. Independently of the academic year, for all the students, this crisis re-enforced their wishes to become nurses:

“I wish I already had my degree” P17; “I wish to be a nurse already, too bad I wasn’t in 4th year so I could go” P16; “If this happens in the future, I would like to be helping” P25; “I feel like left out, I can’t be in the battlefield helping” P21; “Now I really feel like being a nurse. It is a shame that we cannot help. In Granada there is a list of volunteers. I really feel like helping” P14

Master’s students who work feel satisfied to be able to help (aside from being satisfied because they can work):

“I feel very well with myself because I can help, even though is very difficult…” P23; “I really feel like being in the middle of it and help. I’ve seen that help is really needed, it is very important work, although not very much appreciated.” P24

### 3.2. Uncertainty

The lack of concretion about the different aspects related with their studies is mentioned by all the interviewees. This uncertainty is accompanied by unpleasant situations due to the possible outcomes. They are mainly related with matters that could not be resolved relatively fast, such as the clinical practice and the adaptation of evaluation processes:

“We don’t know how they are going to evaluate us. They will for sure evaluate what we have done in the last month of clinical practices” P6; “We don’t know what’s going to happen. I hope they don’t give a general pass. I want to take the exams and the other things. I don’t want them to evaluate me with just one work submitted” P5; “Not knowing how things will be done. Not getting the grades I want to get because of these circumstances” P16

This is especially important for the 4th-year students, who reported a great feeling of wasting time. They cannot go to the clinical practices and they only have, as well, one subject: the final project (TFG). One of the alternatives to not waste time completely and that is being done by the participants is to prepare for the access exam for clinical nurse specialist training (national post-graduate residency program, EIR). Some of the participants indicated that preparing for the EIR exam was a means of escape from a situation of wasting time and total paralysis:

“It takes my motivation away, and (finishing my degree) is getting really hard, because I don’t see the end of it” P10; “I am not taking advantage of the time” P3; “I’m preparing for the EIR exam at the academy as a means of escape. With the only the TFG…I need something else. Right now all my time is TFG and the subjects from the EIR” P4

The 3rd-year students find themselves in the same situation but without any subjects:

“The 3rd years clinical training has been abandoned. They don’t know if we are going to recover them” P6

The Masters’ students have a different point of view. The differences are many. The Masters’ degree can provide job opportunities, the change from traditional education to e-learning practically affects an entire trimester (half of the Master’s program), and in their discourse, they have fewer demands and less pressure for obtaining the degree. At the same time, they are the only ones who speak about the teaching guidelines, indicating that they are truly being followed. In comparison, only one Bachelor’s degree student referred to the teaching guidelines:

“If the clinical training cannot be done, you miss job opportunities” P22; “I don’t know how the teaching guidelines have changed” P16; “The clinical training have been postponed until September, and it bothers me some because it interferes with the summer contract for working as a nurse” P24

### 3.3. Time

Time is a determinant transversal aspect. Two differentiated phases are observed as the state of alarm moved forward (1st shock and 2nd normalization). Besides, participants reflected regarding a necessary time management and the influence in the future.

#### 3.3.1. Phases: 1st Shock and 2nd Normalization

Two well-differentiated phases are distinguished in the timeline. On the first days, the shock phase appears (1st phase), within which we find “disorientation”. This first phase lasts between 7–10 days. During this first week, it is observed that mental performance decreases, along with the ability to concentrate. This is a subtle expectant phase, where the situations are not well defined:

“You think that the first week is for you, for resting, you take care of unfinished business and uncertainty increases” P11; “The first week was not assimilated, I didn’t have routines” P19; “During the first week, I had less concentration and studies less” P5; “The timetable is different, it’s more chaotic” P21

After the first phase, the students enter a normalization phase (2nd phase) in which they acquire new routines, attend online classes and seminars. The conditions of confinement start to be assimilated and the new everyday life is normalized:

“Now I do more things than before, I take more notes. It is very different from the first week, now it is easier” P25; “Now I have the habits. Before I didn’t do anything, and now I do everything, it is as if I’m getting used to it” P28

The first phase, as well as the second phase also coincide with the period in which the university ensured that the online tools were fully functioning and instructions were given to the professors about how to continue with their teaching tasks:

“Only 2 out of 5 teachers give online classes, the rest upload presentations that we have to understand” P13; “The teachers do not agree with each other. One says one thing and another something else” P6

#### 3.3.2. Time Management

The 1st and 2nd year students, as well as the Masters’ students, have classes. This forces them to manage their time differently. The 1st and 2nd year students interviewed indicated that time management was necessary. They indicated that this was beneficial for having good “mental health”, and that having due dates helped them with managing their time:

“Having self-discipline and a timetable. Not rigid, but saying that the mornings were for University and the afternoons for watching T.V. series or exercising. If you don’t organize your time, work accumulates” P20; “My planning is Monday to Friday mornings for work, and the afternoon for group work or leisure. I rest on the weekends. Having due dates has helped me organize” P13; “The homework is good, because they help with following the course” P11

All of the participants, except for the ones who worked, indicated that they had changed their sleep schedule and go to bed much later, between 1:30 and 3 a.m. The main reason mentioned was that the lack of activity did not make them tired, although this argument was ambivalent, as they went to bed later and got up later as well, so they slept the same number of hours:

“It takes me longer to fall sleep. I’m not tired because I don’t do anything during the day” P11; “I go to bed later and I get up later. I go to bed at 2–2:30 a.m. and I get up at 10” P5; “I fall sleep very late. At 2:00 a.m. The hours have changed, you sleep when you shouldn’t” P19

#### 3.3.3. The Future

The participants indicate that this situation affects their future plans and expectations related with obtaining their degree and work. They believe that they can be singled out for being the promotion with missing education, their international training is paralyzed, and they are afraid. Their professional expectations are also affected:

“I’m afraid of having bad training and that the work exchange says that this year’s promotion from the University of Granada do not have the competences necessary” P6; “The plans for earning money to go to an Erasmus program are cancelled…” P13; “The practices have been postponed to September, and yes, it bothers me because it interferes with the summer contract for working as a nurse” P24

### 3.4. Teaching Methodologies

The participants indicate that as for the teachers, different teaching methodologies are being utilized: real-time videoconferences (including chats), lessons recorded on video and uploaded to the e-learning platform, audio podcast, chat (exclusively), homework and uploading of documents (Word, PPT, PDF). They also mention that as time goes on, the teacher’s adaptation to the online resources continuously improve.

#### 3.4.1. Videoconference

It is without a doubt the best evaluated. This is because they think it is the most similar to a traditional class (face-to-face), and allow interaction with the professor, and provides them with nearness. Another aspect they indicate as being valuable is that this methodology helps with the teacher’s explanation of the subject that is more comprehensible as compared to other methodologies. The interaction is also valued, as it allows them to say that something has not been understood and that it should be explained in another way. Lastly, they would like all the videoconferences to be recorded so they could be watched again whenever needed. This last aspect was pointed out by the students who were also working:

“The interaction in the videoconferences is not the same, because the questions are written and it is not the same to write something than when you talk” P26; “The videoconference is where we receive feedback. You can say that you don’t understand something and if it could be explained once again” P16; “It is a way to stay in touch. Doubts emerge and the teacher can resolve them” P7

The Master’s students indicate that on some occasions, the duration of the videoconference classes is excessive. It is interesting to highlight that the Bachelor’s students did not state this at any time:

“We’ve had videoconferences that lasted 5 h. This can be done better. We had one who did a good summary and it lasted 2 h. This is more relaxing, and then you broaden the knowledge with the documents provided” P24

Despite the value of the videoconferences, the discourse is ambivalent, as negative aspects are identified, especially related with the quality of interaction with the professor. The traditional classwork contributes fundamental elements in the quality of communication, and this how it is felt by the participants.

“It is worse. When the teacher sees you asking about a doubt, she/he knows where you are coming from. This is lost with e-learning. Information is lost and the student does not obtain the same information as in the face-to-face class. The teacher doesn’t see your face.” P24; “I’m much more in favor of traditional classes. I always obtain more information in them and I’m more comfortable.” P24

#### 3.4.2. The Rest of the Methodologies

Except for the recorded lessons, the rest of the methodologies are catalogued as sub-standard. The chats (exclusively) and the homework are not attractive, although they value them as positive aspects because it lets them stay connected with the subject and the university:

“The worse thing is when they only upload class notes, no one forces you to read them” P25; “In the homework, there are questions because they are not easy to understand, with the explanation it is easy, but when you are going to do it, it is more difficult” P25

Among the limitations, they point out that in some asynchronous methodologies and with a rigid format, limited learning is obtained, interaction is needed for explanation, and a certain amount of pressure is needed. Another limitation is the lack of feedback with the homework:

“We are going to learn the minimum, but not all, because they don’t explain it to you, they don’t explain it in different ways. The text [from the documents] is only written in one way…” P7; “the works that don’t have feedback give you half the knowledge” P13; “If you only upload notes, no one is forcing you to read them. It is very easy to fall into laziness when they only upload notes” P25

A limitation of e-learning that was pointed out by all the participants was that everything that was practice-related could not be learned. They identified this as a great limitation, and point out that in nursing, practice was vital:

“Many things are not understood through the computer. For example, the basic care laboratories have to be observed and practiced” P7; “The practical things not, but the theoretical yes. They can make a video, but it’s not the same. They can tell us how to give a bath on a bed, but if you don’t do it…” P5; “It is impossible to learn the practical part. Until you are not in that role, it is impossible to learn” P24

The students are not able to propose other methodologies that are distinct from the ones offered. Two students pointed out that it could be completed with gamification (kahoot):

“Gamification would be good, for example when calculating the dose” P19

#### 3.4.3. Use Profile of the Methodologies by the Teachers

Within the methodologies, it was found that the least complex, for example, providing Word, PPT or PDF documents, were related to the older teachers. The videoconferences and recorded classes were given by younger teachers in general. At the same time, they indicated that teachers from other non-nursing departments utilized the least complex methodologies:

“It depends on the difficulty of the course. Physiology has only uploaded documents” P16; For example, Pharmacology is a very dense and complicated subject, and you need someone to explain it to you, and until now, we have not received anything, only notes. I don’t think it’s enough, they are too schematic and hard to understand”. P1; “The younger ones (teachers) feel like doing more things” P19; “It is more difficult for some teacher, especially those who are older” P5

#### 3.4.4. Interaction with the Teachers

They pointed out that it is good in the videoconferences. An inconvenience is that sometimes the teacher is not aware of the doubts posted on the chat if there are too many messages. In the chat, the interaction is good, but the interruptions, even though they may be short, makes it impossible to follow it. Lastly, the students are surprised about how fast the teachers answers the e-mails:

“The chat, if you miss 5 min, you get lost” P22; “There is a good reception by the teacher for communicating” P11; “[tutoring} they are good, the answer sooner. They have improved” P21

### 3.5. The Context of Confinement and the Added Difficulties

The context of confinement has created some limitations for following e-learning education. These are related with internet access, access to electronic devices, and work and family responsibilities.

In rural environments, situations exist where internet access is lacking, which creates problems with being up to date with the classes. Another problem indicated is that not all had internet at home, and situations exist in which a person only has the limited amount of data available from a smartphone:

“Some people do not have all the means” P25; “I don’t have internet at home, I only have data from the smartphone” P27 “I live here in the countryside, and the internet does not always work well, and if my kids are connected, then I can’t do anything” P17

The confinement has obliged working from home whenever possible. This implies that it is possible for a family with three children to need an internet connection at the same time and the availability of five electronic devices simultaneously to be able to work and follow the classes. This availability is not very common. Another limitation that was pointed out was working in the presence of children/siblings at home:

“With the children at home, things cannot be done [mothers]” P25; “Studying at home when the entire family is at home, it is very hard to concentrate sometimes, they make noise, I can’t print, etc.” P25

Part of the students pointed out that is inconvenient, as they are used to studying in public libraries and have had to study at home:

“I always study at the library, not at home” P19; “I used to go to the library to study or do homework. No one bothers me there. At home, I set the washer, put on my pajamas and go to sleep” P28

Another difficulty added by the confinement is that one is not “trained” for shifting to e-learning. One has experience with an education system that has never been 100% online and where the traditional class is the learning stage. With respect to online exams, they do not feel secure either:

“We are used to traditional classes. This has been difficult for everyone, and more for the bachelor students than the Masters ones” P23; “I supposed they will give multiple-choice exams in a short time. It is the first time it will be online and one could be tense” P13; “If I hear it from the teacher beforehand, I understand it better, and now it’s different. You take notes and then you have to understand them…” P16

### 3.6. Face-to-Face Win

#### 3.6.1. Face-to-Face is Better…for Everything

The participants clearly preferred face-to-face to e-learning education. When faced with the possibility that some percentage of online classes will be provided along with traditional classes in future academic years, they do not think it is an option that will contribute much or needed. An exception is provided by students who have family or work responsibilities, who, exclusively for the theoretical classes, prefer them to be online and recorded, in order to be able to watch them at any time. Another aspect that was underlined was that the traditional system of education is the one they know and are used to, and changing it is difficult:

“Face-to-face is better…for everything” P17; “The University of Murcia is traditional, and we come from the same type of learning. It takes some time to adapt” P22; “Face-to-face is better in every aspect. For example, you learn the lesson and the teacher can provide examples, it can go further than the PowerPoint presentation. It is better to be face-to-face with the professor than through a screen” P23

#### 3.6.2. Older and Female: Face-to-Face is Better

The older students seem to be the most vulnerable group, and various problems are observed. On the one hand, they have to tend to their children now that they are all at home, they have more responsibilities at home, plus certain digital competences that they have yet to incorporate. The management of their time is a great problem, which is influenced by the use of time, space and the electronic devices by the rest of the family, to which they grant them priority without being aware:

“For me the chat is not good, because I can’t write that fast. I see the limitation in me. I miss the traditional classes. Face-to-face classes are better…for everything” P17; “You have to be very alert with the online classroom, that you do not ignore the messages. Yesterday there was a class, and did not know” P9; “Some classmates are much older, and this is difficult. They write to the group [WhatsApp] sending pictures, and asking “What do I do? Where should I click?”” P25; “I’m much more in favor of face-to-face classes. I always obtain more information in them and I’m more comfortable” P24

## 4. Discussions

It is necessary to underline that all the results and discussion are centered on the first month of confinement after the start of the state of alarm, and this brings with it very specific cognitive and social states that are needed for the proper understanding of the discussion of the present research study.

Although the sample included a greater number of students from the University of Murcia as compared to those from the University of Granada, and different percentages of men and women of different ages, we believe that the main sociodemographic variables were well represented through the use of maximum variation sampling.

The nurses usually become nurses due to their desire to help other people to recover and maintain optimal health, and here we find ourselves in a situation in which not many options are available to help those who are severely sick due to COVID-19 [5]. Vocation is a determinant factor for those who decided to study nursing, and the main drive is the opportunity to care for others [17]. Our results support these two ideas in two ways: (1) they indicate that this attitude towards their professional life is still true in the new generations, with the remarkable fact that all the participants are so committed and wishing to help. (2) the pandemic has positively re-enforced their wishes to become nurses, obtaining similar results as other authors [18]. Although, the state of alarm decree includes the possible mobilization of students in their last year at university, their mobilization was principally needed in a small scale in Madrid and Catalonia, the areas greatly affected by COVID-19 [19].

The fast shift to e-learning education has not ceased to be a continuation of teaching and education through online resources, although it has not been clearly planned and adapted for e-learning [10]. Our results clearly present various relevant ideas related to this. In first place, and related with the clinical training, the health science degrees and more specifically the nursing degree have an essential need to be developed in clinical context. This element clearly cannot be substituted, and is perceived by all the students as being essential. Nevertheless, at present, a discussion exists about how high-fidelity clinical simulation could substitute the clinical training in real-world environments [20,21]. This methodology, which facilitates an intermediate learning between the theoretical dimension and the practical dimension, is proposed, aspiring to construct a real environment. However, and despite it being a type of learning established and known by the student body at the University of Murcia as well as the Granada, it is striking that this type of learning has not been described as an alternative. We interpret this finding as the clinical training being indispensable for the students. 

Also, it forces us to reflect if this is the new reality of health care, and if the future nursing professionals should learn how to navigate in these conditions. The debate regarding the return of the nursing students to clinical environments is open and some recommendation has been provided [22]. The question now for the universities and nursing educators is that if as soon as the resources are provided and an adequate organization and adaptation occurs, the students should return to the health care services, what is the balance between the potential risk for the students and the importance of the clinical training? In second place, and related with the teaching of theory, the students prefer face-to-face teaching as opposed to the e-learning. They believe that the interaction is higher in quality and learning is greater. At present, another debate is open, as shown by two systematic reviews that do not provide concluding results on the existence of the greater learning linked to e-learning education of health professionals and students, highlighting the poor quality of existing studies and the importance of contextual factors [23,24]. Perhaps due to these reasons, the videoconference, distance learning, but synchronous and bi-directional, is the best assessed.

Another critical aspect is that the change to the online methodology was not chosen by the students, and the expectations they have with respect to their studies have been clearly disrupted. Their entire academic life has been marked by a specific style of teaching, and they have become organized to continue with it, but the pandemic has imposed a different one with which they do not feel comfortable yet, thereby creating uncertainty and little security. This worries a great part of the Health Science academics [25]. It therefore absolutely necessary to start to work on the adaptation to e-learning that takes into account the previously-mentioned aspects so that the student’s uncertainty decreases, especially in light of the evaluations. Academics have already expressed awareness of the students’ concerns that are centered on their future degree and career progression [25]. The university counted with a technological infrastructure that has been able to deal with a drastic and fast change to distance teaching. However, the urgency of adapting this type of teaching has highlighted some situations of disadvantage. Thus, the older students, as compared to the younger ones, and in great part women and mothers, do not possess the most basic digital competences. This finding is robust, as the older students themselves, as well as the younger ones, are able to point this out in agreement with each other. They also point out that there is a small percentage of students who do not have the electronic resources or a connection to the internet necessary for adequately following the teaching.

Universities are trying to provide answers to some of these problems. It could be said that the phases of shock and normalization described by the students coincided with the period of reaction and acts of implementation by the institutions. There are activities that allow for fast implementation. For example, the Universities of Murcia and Granada freely loaned laptop computers with software to 100% of the students who requested them [26], with this number being more than 300 students in Murcia alone, as well as mobile internet-access devices [27]. However, the implementation of activities related with the evaluation has required conscientious reflection and consensus that has forced their implementation later in time [11].

In any case, once this first stage has been overcome, and faced with the absence of permanent solutions for this pandemic in the short term, it is necessary to propose distance learning strategies with a robust design, with the time necessary to create study plans that are well thought-out and durable [10,28]. We should be aware that we are currently undergoing an “emergency” education, a temporary shift of instructional delivery to an alternate delivery mode due to crisis circumstances [29]. The reality is that this transition to e-learning under these circumstances has nothing to do with a design that takes the maximum advantage and possibilities of the online format. We should reflect on the differences in the rhythm, the student-instructor relationship, pedagogy, the role of the instructor, the role of the student, the synchronicity of online communication, the role of online evaluations, and the source of feedback [30].

### Limitations

Among the limitations of the study, we find that a thorough discussion and comparison with the opinions of other authors has not been possible, given the novel and exceptional situation we are currently living in. On the other hand, we should be aware that the sample studied cannot be representative of the reference population, and this can evidently affect the generalization of the results.

## 5. Conclusions

After the first week of adaptation to the conditions of confinement and the establishment of new online teaching systems, the students begin a new normality. The imposition of e-learning brings more limitations to students who are older, with work and family responsibilities, living in a rural environment and with limited electronic resources. Online teaching has allowed substituting the teaching of theory, although face-to-face teaching is preferred, at the same time it has shown that clinical practices are indispensable for the training of the nursing students. Online education goes beyond the online continuation of the classes. The parties responsible should already be working on this for the next academic year, in light of the uncertain future of a short-term control of COVID-19.

## Figures and Tables

**Table 1:** Characteristics of the participants and duration of the interviews.

N°	Gender	Age	University	Degree Year	Working	Interview Duration
1	Woman	20	Murcia	2	No	39 min
2	Woman	19	Murcia	2	No	50 min
3	Woman	21	Murcia	4	No	20 min
4	Woman	21	Murcia	4	No	20 min
5	Woman	28	Granada	1	Yes	38 min
6	Man	21	Granada	3	No	20 min
7	Woman	19	Granada	1	No	36 min
8	Woman	41	Murcia	1	Yes	38 min
9	Woman	50	Murcia	3	No	35 min
10	Woman	21	Granada	4	No	23 min
11	Woman	20	Murcia	2	No	51 min
12	Man	32	Murcia	4	Yes	23 min
13	Woman	19	Granada	2	No	37 min
14	Woman	22	Granada	4	No	19 min
15	Man	21	Murcia	4	No	17 min
16	Woman	20	Granada	1	No	28 min
17	Woman	46	Murcia	2	No	40 min
18	Woman	25	Murcia	3	No	31 min
19	Woman	20	Murcia	3	No	31 min
20	Woman	25	Murcia	1	Yes	29 min
21	Woman	26	Murcia	4	No	18 min
22	Man	24	Murcia	Master	Yes	25 min
23	Woman	23	Granada	Master	Yes	28 min
24	Man	22	Granada	Master	No	29 min
25	Woman	18	Murcia	1	No	33 min
26	Man	42	Murcia	3	No	29 min
27	Man	26	Murcia	3	No	30 min
28	Woman	24	Murcia	Master	Yes	30 min
29	Man	26	Murcia	3	No	30 min
30	Woman	20	Murcia	2	No	35 min
31	Woman	24	Granada	Master	No	26 min
32	Woman	26	Murcia	Master	No	36 min

**Table 2:** Themes and subthemes.

Themes	Subthemes
Practicing the nursing care	The value of clinical training
	To help
Uncertainty	-
Time	Phases: 1st shock and 2nd normalization
	Time management
	The future
Teaching methodologies	Videoconference
	The rest of the methodologies
	Use profile of the methodologies by the professors
	Interaction with the teachers
The context of confinement and the added difficulties	-
Face-to-face win	Face-to-face is better…for everything
	Older and female: face-to-face is better

## Appendix A.

**Table A1:** Interviews Guide.

Introductory questions. How is your…?	- Leisure - Sleep - Rest - Diet - Exercise - Interrelation - Information about COVID19, where they are informed, if they have reviewed notes of any subject (handwashing, concept of prevalence, incidence...).
Online teaching methodology	- M1 Do you think that the university is providing an adequate response to the situation related to teaching? - M2 What types of tools are being proposed to you. - M3 Which one do you like best, describe advantages and disadvantages. - M4 What things/competencies cannot be learned (teaching limitations). - M5 Is it difficult to adapt to the communication channels that are used right now? - M6 Is the content given in class adequately adapted to online teaching methodologies? - M7 How is your interaction with other participants different from face-to-face? - M8 How is your interaction with the teacher? - M9 Tasks
Learning	- A1 How is time organized with this imposition - A2 Has your ability to concentrate and/or study been affected? - A3 What are the differences when studying in this manner? - A4 Do you think that this manner of learning forces a different type of learning management? Is it necessary to have more self-management? - A5 Is it possible to achieve learning in the same way as face-to-face learning, better, worse... - A6 What modalities, online methodologies do you propose for improving your online learning?
Expectations	- E1 Expectations about grades - E2 What influence do you think this way of teaching during this time will have on your training as a nurse. - E3 Do you think that after this online teaching a greater part of it will be necessary when everything returns to normal? Why? - E4 Are students prepared for this type of teaching? - E5 Do you think it is necessary to go one step further? Should this type of teaching go further?
4th year students	- AA1 What is going to happen? - AA2 What are you afraid of? - AA3 What do you think will happen with the practice? - AA4 What do you think will happen to your degree? - AA5 If you miss a practicum how good is your training?
Life expectancy	- How does the situation caused by the crisis in the professional field affect your desire to become a nurse?
